# Quantitatively evaluate the impact of domestic aviation control measures on the spread of COVID-19 in China

**DOI:** 10.1038/s41598-022-21355-5

**Published:** 2022-10-20

**Authors:** Yu Wang, Ke Li, Ting Yuan, Yi Liu

**Affiliations:** 1 Center for Applied Statistics, Renmin University of China, Beijing, 100872 China; 2 School of Statistics, Renmin University of China, Beijing, 100872 China; 3 Key Laboratory of Civil Aviation Data Governance and Decision Optimization, Civil Aviation Management Institute of China, Beijing, 100102 China

**Keywords:** Diseases, Infectious diseases

## Abstract

To quantitatively evaluate the impact of domestic aviation control measures on the spread of COVID-19 in China. The number of international flights from March to September 2019 simulated the number of flights from March to September 2020 without implementing aviation control measures. In addition, the proportion of asymptomatic persons and the delay in case reporting were adjusted to estimate the prevalence of each country during the same period and calculate the estimated imported cases. The estimated imported cases were assigned each day with weight, and the estimated daily reported cases were obtained based on the actual daily number of domestic cases in China. Effective Reproduction Number ($R_t$) was calculated based on delayed distribution, Basic Reproductive Number ($R_0$) distribution, and generation time distribution were reported in previous studies. Gaussian Process was used to estimate the effect of time-varying on $R_t$, and the estimated $R_t$ was compared with the actual $R_t$. The estimated imported cases increased significantly compared with the actual number of imported cases. The estimated imported cases were mainly concentrated in North America and Europe from March to April and gradually increased in many East Asian countries from May to September. The difference between predicted $R_t$ and actual $R_t$ was statistically significant. The estimated imported cases and the estimated $R_t$ have increased compared to the actual situation. This paper quantitatively proves that Chinese aviation control measures significantly suppress the COVID-19 epidemic, which is conducive to promoting and applying this measure.

## Introduction

At the end of 2019, Corona Virus Disease 2019, also called COVID-19, spread through the whole world via carriage and infection from person to person. On March 22nd, 2020, it was declared a pandemic by the World health organization. The clinical characteristics of which contained high speed of infectivity and prolonged incubation. As of June 15th, 2021, COVID-19 cases had reached 175,847,347, including 3,807,276 death cases^1^. Every country has implemented the corresponding control measures to cope with the increasing access to health care^2^. The United Nations World Tourism Organization reported that, as of April 20th, 2020, nearly every country had implemented some form of COVID-19-related travel restrictions^3–5^ . This was the most extensive travel restriction throughout history^1^, involving closing the border, suspending the flight, and implementing isolation and self-quarantine for the tourists.

In preventing and controlling the domestic epidemic of COVID-19 in China, the government decisively adopted a series of prevention, control, and treatment measures. It took one month to prevent the spread of the epidemic and about two months to maintaining the daily new cases in the mainland within single digits. It took about three months to cease the spread of the disease in Wuhan, Hubei province. At the same time, the epidemic abroad gradually developed and reached a dire situation. “External defense input and internal defense rebound” has become the dominant strategy in China. In this situation, the Civil Aviation Administration of China (CAAC) put forward a “Five-one” policy, that is, the international flight policy of “one airline, one country only reserves one route, and executes at most one flight a week” implemented since March 29th, 2020. After implementing the “Five one” policy, 11 domestic and 95 foreign airlines suspended international passenger flights to China. Compared with the situation that 30 domestic and 123 foreign airlines operated international passenger flights before this strategy. Further prevention measures included diversion and whole process management at the first entry point and nucleic acid testing at all ports.

Due to the travel restrictions implemented by some regions during the epidemic, international aviation has shown an unprecedented downturn. The operation of China’s international flights in 2020 showed a steep decline compared with that in 2019, which was the direct result of the measures of aviation control and prevention the importation from outside. The studies of Zhang et al.^6^ and Bielecki et al.^7^ showed that travel restrictions have apparent effects when the cases in the destination country are zero or less. When the number of cases imported from international tourists dramatically impacts the total number of new cases, measures such as travel restrictions or blockades will be effective^8^ . Therefore, it is complicated to evaluate the effect of travel restrictions on curbing the spread of COVID-19. It is necessary to consider the local epidemic spread in importing countries and the travel volume from these countries to China. At the same time, it is also essential to evaluate the extent to which imported cases have affected the Reproduction Number ($R_t$). No research quantitatively analyzed aviation controlling measurements’ effect on domestic infection until now. This study used the number of international flights from March to September 2019 to simulate the operation of flights without flight restrictions from March to September 2020. At the same time, it will use the epidemic data of COVID-19 worldwide from March to September 2020 to estimate the expected imported cases without travel restrictions and further evaluate the impact of the expected imported cases on $R_t$ in China.

### Contribution

1. Estimate the expected imported cases without travel restrictions from March to September 2020.
2. Further evaluate the impact of the expected imported cases on $R_t$ in China.
3. Help develop similar public health response plans in the future.

## Methods

In this study, we used the method proposed by Russel^8^ to estimate the actual number of COVID-19 cases and calculated the prevalence rate in countries around the world from March to September 2020. We used detailed international flight data in each country from March to September 2019 to simulate the flights from March to September 2020 if no airline restrictions were taken. The expected number of imported cases was estimated from March to September 2020 if there were no airline restrictions. We evaluated the predicted number of imported cases in the major importing countries from March to September 2020. The impact of airline restrictions on the number of domestic time-varying $R_t$ was estimated to measure the extent to which the airline restrictions implemented in March 2020 had controlled the development of the COVID-19 progress in China.

Because there were a substantial number of asymptomatic infections^9,10^, and in addition there were delays in reporting cases^11^, the available data on reported cases may not reflect the actual number of infections in the local area at that time. It was necessary to use mathematical models to adjust the reported cases to obtain the actual infection situation. First, the level of case ascertainment in each country was estimated as the ratio of a delay-adjusted country-specific case fatality ratio to an assumed baseline case fatality ratio (derived from published estimates). Then, temporal variation in under-ascertainment was inferred using a Gaussian process: a non-parametric Bayesian framework, suited for statistically robust estimates of time-dependent functions. Finally, these temporal under-ascertainment estimates were used to adjust the confirmed case time series. The adjusted case data represent the estimated true number of symptomatic individuals in each country, which is typically substantially larger than the confirmed case number.

### Calculation and adjustment of prevalence rate in the importing country

#### The delay between the confirmation of a case and the death

Since it could take 2–3 weeks from the onset of symptoms in a person to the subsequent detection and reporting of a case and observation of the final clinical outcome^12^, and also the result of some confirmed cases cannot be confirmed during an expanding epidemic transmission situation. In other words, the interval from the onset of symptoms in a case to the outcome had a censoring effect; the distribution of temporal interval from the beginning of actual symptoms to the outcome was biased. So simply dividing the cumulative number of reported deaths by the cumulative number of reported cases would underestimate the Case Fatality Rate (CFR) in the early epidemic period^13^. The actual distribution was usually longer in duration than the distribution fitted to data that included censored cases^14^. This pattern had also appeared in CFR estimates for previously prevalent respiratory infectious diseases, including severe acute respiratory syndrome (SARS)^15^ and H1N1 influenza^16^. On the other hand, the CFR of COVID-19 collected by hospitals and the CDC usually focused on critical cases^17^, and the CFR calculated in this way was not applicable to describe the mortality of the entire infected population. Therefore, estimating the number of undiagnosed cases was necessary to know the actual number of patients under the accurate distribution.

#### Inference of undiagnosed cases

The COVID-19 cases consisted of both reported cases and unreported symptomatic cases. The unreported cases are calculated^14^ using the delay distribution from case diagnosis to death to estimate how many diagnosed cases had not observed definitive outcomes (including death and recovery). Thus the Adjusted Case Fatality Rate (dCFR) is estimated for each country. The ratio of the fatality rate, which served as the baseline fatality rate (bCFR) calculated by the available death data to the estimated dCFR for a given country, could be used to estimate the proportion of reported cases to the total number of cases in that country roughly^18^. The specific calculation procedure was as follows.

Assuming $\mathrm{dC}_{c,t}$ as the proportion of the cases with a known outcome in c country on the t day. The formula was as follows:

1 $$\begin{aligned} \mathrm{dC}_{c,t}=\sum _{j=0}^{t}{C_{c,t-j}g_j} \end{aligned}$$

Where $\mathrm{C}_{c,t}$ represented the incidence of COVID-19 in the c country on day t; $g_t$ was the proportion of cases with known outcomes at time t. Its distribution represented a probability mass function of the proportion of cases with available outcomes at the time interval from diagnosis to death. In this study, we used log-normal distribution to fit the time interval from diagnosis to death using data related to cases from hospitalization to death in the literature^19^ to obtain a mean value of 13 days (95% CI 8.7–20.9 days) with a standard deviation of 12.7 days (95% CI 6.4–26 days).

Let $\mathrm{dCFR}_{c,t}$ be the delay-adjusted case fatality rate on day t in c country to estimate the proportion of confirmed cases with known outcome events. The following equation was therefore obtained:

2 $$\begin{aligned} {\mathrm {dCFR}}_{c,t}=\frac{D_{c,t}}{\mathrm{dC}_{c,t}} \end{aligned}$$

Where $D_{c,t}$ was the number of fatal cases on day t in c country. Let $a_{c,t}$ be the proportion of confirmed symptomatic cases on day t in c country, and the formula was as follows:

3 $$\begin{aligned} a_{c,t}=\frac{\mathrm {bCFR} }{{\mathrm {dCFR}}_{c,t}} \end{aligned}$$

where bCFR was set as a base value of 1.4% (95% CI 1.2–1.5%). It was a constant value across countries concerning a study of COVID-19 CFR in China^12^. And based on constructing the framework as simple and easy as possible, bCFR for each country was adjusted by age distribution to prevent the population’s age distribution in each studied country from being different from that of China^14^. The estimation of $a_{c,t}$ was a pointwise estimate that did not have stability over time^20^, thus a Gaussian process was adopted to fit the situation of how $a_{c,t}$ varies over time.

Let $a_{c,t}^*$ be the proportion of identified symptomatic cases that vary over time (determination rate), and the following equation is given:

4 $$\begin{aligned}&\Phi ^{-1}\left( a_{c,t}^*\right) =f_c\left( t\right) +\epsilon _{c,t} \end{aligned}$$

5 $$\begin{aligned}&\varepsilon _{c,t}\sim N\left( 0,\sigma _{c,1}^2\right) \end{aligned}$$

where $f_c(t)$ was the nonparametric equation of the country c concerning time t, $\varepsilon _{c,t}$ were mutually independent random variables, $\Phi ^{-1}$ was the inverse of the probit function, which allowed mapping the function values to the range of the determination rate per unit of time;$\ f_c(t)$ will be set as the following Gaussian process equation:

6 $$\begin{aligned} f_c\left( t\right) \sim GP\left( 0,k\left( t,t^\prime ;\theta _c\right) \right) \end{aligned}$$

Where $k(t,t^\prime ;\theta _c)$ was the covariance function, which consisted of two parts of the covariance matrix:

7 $$\begin{aligned}&k_{bias}\left( t,t^\prime \right) =\sigma _{c,2}^2 \end{aligned}$$

8 $$\begin{aligned}&k_{SE}\left( t,t^\prime \right) =\sigma _{c,3}^2e^{-\frac{1}{2l_c^2}|t-t^\prime |^2} \end{aligned}$$

$k_{bias}$ was called the deviation kernel function and was used to construct the mean of $a_{c,t}$ throughout the time interval. $k_{SE}$ was called the squared exponential covariance function and was used to determine how this mean value varied over time.

#### Adjusting for prevalence

Monthly prevalence in importing countries was estimated using the method of Russell^8^. Publicly available data from the European Centre for Disease Control (ECDC), including time-series data on daily new cases (${Case_{c,t}}$) and deaths ($D_{c,t}$) in each country, and the average population in each country ($P_c$) was used to estimate daily cases with uncertain outcomes in each country. We selected the countries with at least ten deaths per day and more than ten days to generate deaths to obtain $a_{c,t}$. The proportion of asymptomatic infections (${ASY}_{c,t}$) that vary with age has been confirmed^21^. But in the absence of age-stratified data in globally, we assumed to set ${ASY}_{c,t}$ as a constant across countries using the levels reported in the literature^11^, a mean of which was 50% (95% CI 10–70%). We adjusted the ${Case}_{c,t}$ in the two above aspects to obtain $a{Case}_{c,t}$ and then the number of cases was summed monthly to obtain the estimated whole number of cases ($a{Case}_{c,T}$) per month for each country:

9 $$\begin{aligned}&a{Case}_{c,t}=\frac{Case_{c,t}}{{a_{c,t}}{(1-ASY_{c,t})}} \end{aligned}$$

10 $$\begin{aligned}&a{Case}_{c,T}=\sum {a{Case}_{c,t}} \end{aligned}$$

Combined $P_c$ to obtain the adjusted prevalence for each country per month (${Pre}_{c,T}$).

11 $$\begin{aligned} {Pre}_{c,T}=\frac{a{Case}_{c,T}}{P_c} \end{aligned}$$

In addition, considering the uncertainty of the estimates, we used ${ASY}_{c,t}$ and bCFR values and ranges to derive 95% confidence intervals for the final adjusted cases.

### Flight between importing countries and China

The data were obtained from the IATA flight data between countries worldwide from March to September 2019. They were used to simulate the flights between March and September 2020 without flight restrictions and controls, and all restricted flights were from abroad to China, excluding flights within the country. The flight data included the month of the flight, the country of origin and destination, and the number of flights to obtain the expected monthly airline data from March to September 2020 from each country to China($Airport_{c,T}$).

**Figure 1:**
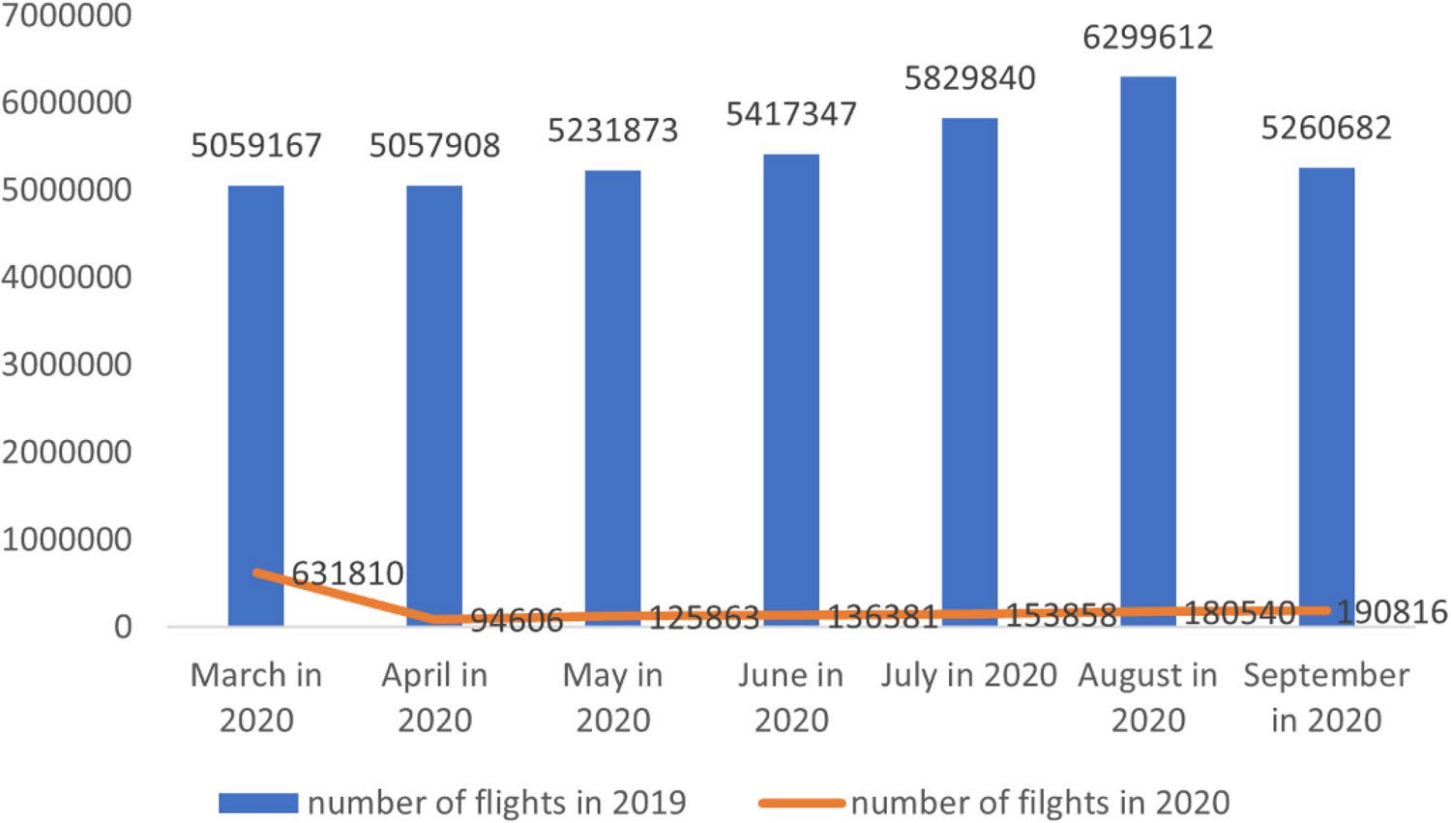
Total number of flights from March to September in 2019 and 2020.

Based on the data provided, it can be seen that the number of flights in 2020 was significantly lower compared to the same period in 2019 due to the COVID-19 epidemic and the implementation of related aviation controls, as shown in Fig. 1.

### Expected imported cases

Based on the above estimates of the prevalence of COVID-19 in each importing country, the prevalence rate in each importing country from March to September 2020 was obtained. The expected number of imported cases per month in each importing country without controls ($Import_{c,T}$)was equal to the adjusted prevalence estimates ($Pre_{c,T}$) for each importing country each month from March to September 2020 multiplied by the number of flights passengers ($Airport_{c,T}$) from each country each month from March to September 2019:

12 $$\begin{aligned} {Import_{c,T}}={Airport_{c,T}}{Pre_{c,T}} \end{aligned}$$

### Estimation of the effective reproduction number

The role of flight control in the pandemic control process was quantified by comparing the domestic time-varying $R_t$ calculated by the actual reported cases and the time-varying $R_t$ calculated by the expected importing cases in the absence of flight control. Abbott^22^ proposed the method for calculating the time-varying $R_t$. Using the calculated daily estimated reported cases ($eCase_t$) as the initial number of infections ($R_0$), a predictive model was used to infer the most likely infection time given the case confirmation date. Bayesian inference of the possible time of infection was made using a deconvolution approach within a Gaussian process framework, thus providing the database for the estimation for $R_t$.

#### Data source

The $eCase_t$ in our country per day without air traffic control was to be calculated. The estimated monthly number of imported cases ($Import_T$) was assigned each day using the distribution of reported cases in the actual situation. Here we set the weights as follows:

13 $$\begin{aligned} weight=\frac{Case_t}{Case_T} \end{aligned}$$

Then the estimated number of imported cases ($Import_t$) in our country per day was calculated as follows:

14 $$\begin{aligned} Import_t={Import_T}{weight} \end{aligned}$$

The $eCase_t$ was the sum of the estimated number of imported cases and actual reported cases each day:

15 $$\begin{aligned} eCase_t=Import_t+Case_t \end{aligned}$$

#### Delay in reporting cases

Because of the temporal delay between patient infection and case reporting, it was necessary to estimate the reporting delay time with some uncertainty and thus measure the impact of this delay on the number of reported cases. Log-normal distribution of delay times was first simulated, and then 100 re-samples were performed to calculate the mean of delay time.

**Figure 2:**
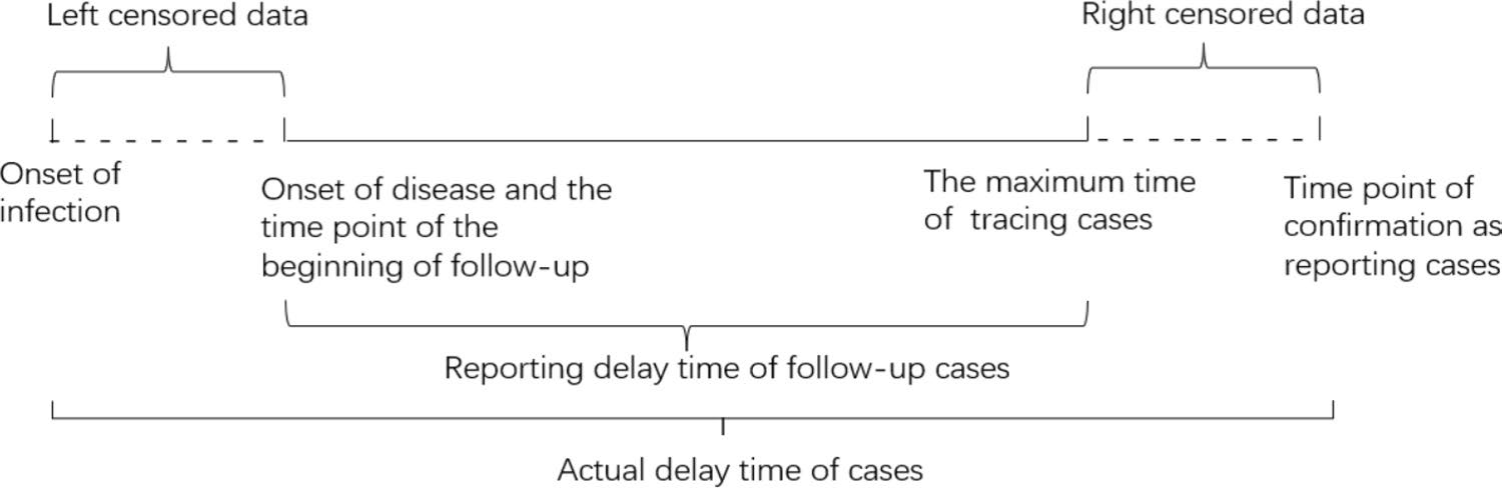
Reporting delay time of cases.

The left and right-censored times were replaced with the most recent date that could be tracked, and the case reporting delay time was truncated at the maximum observable value, as shown in Fig. 2.

Since there were not enough data to study the variability of case reporting delay time, we assumed that such delays were fixed. We made use of the mean value of reported case delay published in the literature of 6.5 days with a standard deviation of 17 days, while the mean value of onset to death reporting delay was 13.1 days with a standard deviation of 11.7 days, and the maximum delay was set at 30 days.

#### Basic reproductive number

The initial reproductive number was set using a log-normal prior with a mean of 1 and a standard deviation of 1. This setting was based on the current situation in most parts of the world, where public health interventions and individual behaviors were used to prevent significant increases in reproductive numbers over time.

#### Generation time

**Figure 3:**
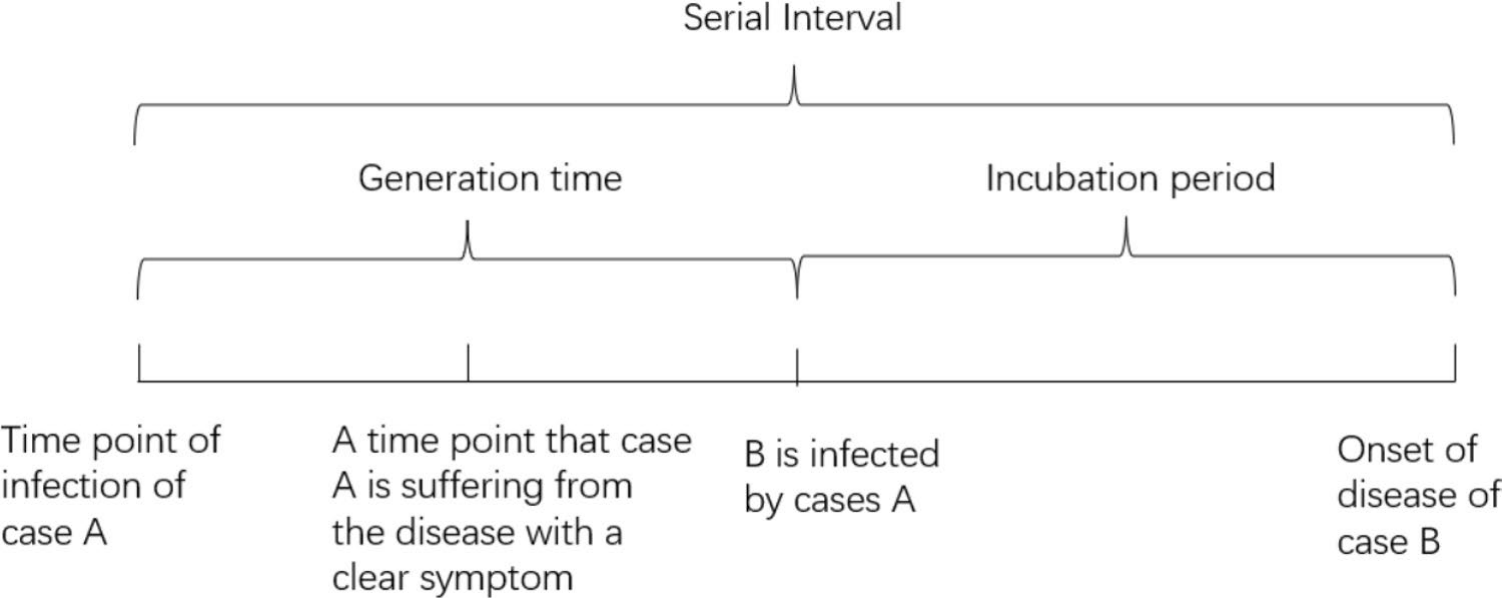
The time interval between the onset of symptoms in different generations (serial interval).

We used the generation time to predict when the transmission of COVID-19 began and to measure the infectiousness of this virus. Generation time describes the interval from when a patient was infected to when that patient transmitted to the next person. However, generation time was challenging to obtain in reality, so we used the time interval between the onset of symptoms in different generations (Serial Interval) instead of the generation time^23^. The relationship between these two is shown in the following figure (Fig. 3).

The time interval between the onset of symptoms in different generations differed from the generation time in that the former included the incubation period of the infected person. Here we used a log-normal distribution to estimate the incubation period. We set the mean incubation period to 5.1 days (95% CI 4.5–5.8), as described in Lauer^12^.

#### Estimating time-varying $R_t$

$R_t$ represented the average number of the second generation confirmed cases that a particular infected person diagnosed at a given moment t will transmit during their infection period. It can be used to measure the real-time transmissibility of an epidemic. When $R_t>1$, an epidemic will spread rapidly through the population, and as the value gradually decreases, the speed of development of the epidemic will also gradually decrease; when $R_t<1$, the number of infections will gradually decrease, and the epidemic will slowly die out.

The EpiNow2 package was used to estimate time-varying $R_t$ based on the date of infection^22^. The corresponding infection time was inferred from the incubation and reporting delays distribution and reported cases’ periodicity. Where the actual time-varying $R_t$ was calculated using real domestic reported cases, the expected daily domestic reported cases were the sum of the daily expected imported cases and daily indigenous cases introduced in the previous section. We used the EpiNow2 program package to calculate the time-varying $R_t$, which can’t be observed directly. So we used a Bayesian latent variable approach, which we can infer through a mathematical model using observed variables. The model works as follows.

The initial number of infections was estimated based on the initial number of cases as a priori. For calculating the infection rate $$I_t $$at moment t, the number of infections estimated before t was weighted by the probability mass function (w) of the first generation case’s generation time and then accumulated, then multiplied by the estimate of $$R_t$$.

16 $$\begin{aligned} I_t={R_t}{\sum _{\begin{array}{c} \varvec{\tau } \end{array}} {w_{\varvec{\tau }} I_{t-{\varvec{\tau }}}}} \end{aligned}$$

Using a log-normal distribution with mean one and standard deviation one as the prior distribution of the $R_0$. Then, convolving the incubation and reported delayed distributions of infected cases (convolved into $\varvec{\xi }$) at each time step, the resulting infection trajectory was expressed as the mean number of reported cases ($D_t$).

17 $$\begin{aligned} D_t={\sum _{\begin{array}{c} \varvec{\tau } \end{array}}}{\varvec{\xi }}_{\varvec{\tau }}{I_{t-{\varvec{\tau }}}} \end{aligned}$$

We assumed that the observed number of reported cases ($C_t$) was generated by a negative binomial observation model with a dispersion of $\phi$ ($\phi$ using an exponential prior with a mean of 1). The mean was the average number of reported cases multiplied by the impact of a particular day of the week, where the independent parameter (${\omega }_{tmod7}$) for each day was used to measure the effects of that day.

18 $$\begin{aligned} {C_t}\sim {NB(D_t {\omega }_{tmod7},\phi )} \end{aligned}$$

The time-varying of $R_t$’s was controlled by an approximate Gaussian process with a squared exponential kernel. The parameters of the Gaussian process kernel function were estimated using the following priors: an inverse gamma prior was used for the length scale, and a standard normal distribution was used for the magnitude. A Markov chain Monte Carlo method (MCMC) was used to fit each time series independently. Here we used four chains with 500 preliminaries each and 4000 samples after preliminaries. Convergence was assessed using R hat diagnostics.

19 $$\begin{aligned} R_t\sim {R_{t-1}\times GP} \end{aligned}$$

where GP was the kernel function of the Gaussian process, $I_{t-\tau }$was the infection rate before the moment t, and $w_\tau$ was the value of the probability mass function (w) of the generation time of the first generation cases at the moment $\tau$.

## Results

### Actual and adjusted prevalence rates for each critical importing country

The actual prevalence estimates of each critical importing country were shown in Table 1, and the prevalence rates of each country fluctuated significantly or slightly from March to September 2020.

Among the critical importing countries with significant fluctuations in prevalence, such as Belgium, Spain, Italy, the United States, the United Kingdom, the Netherlands, Germany, Iran, and Russia, most of them showed an increasing and then decreasing trend in prevalence from March to June. The United States and Singapore were exceptions. The prevalence rate in the United States showed an increasing trend from May to June; the prevalence rate in Singapore showed an increasing and then decreasing trend from April to June, and the rate of decrease was faster than the increase.

Between June and September, the second pandemic of COVID-19 began in some countries, such as the United States, Spain, France, Belgium, the Netherlands, the United Kingdom, and Canada. Specifically, the U.S. showed a second increase in prevalence from May and a significant increase in June, peaking in July; this far exceeded the prevalence rate in the first pandemic and the prevalence rate in the rest of the countries during the same period. Meanwhile, other countries showed an increasing trend in prevalence since June. For example, Spain conducted a growing trend in prevalence rate since June, which increased significantly in July and continued to increase until the month of our study, considerably exceeding the prevalence level during the first pandemic. In France, Belgium, the Netherlands, the United Kingdom, and Canada, the increase in prevalence rate during the second pandemic was relatively low. It did not exceed the level of prevalence during the first pandemic, but the upward trend was still more pronounced. At the same time, the prevalence rate in some countries was decreasing or slightly fluctuating, such as Singapore, Russia, and Iran.

We adjusted the number of currently reported cases considering the proportion of subjects with symptoms still undiagnosed and asymptomatic infections^12^, and the delay in case detection was deemed. The adjusted prevalence rates for each critical importing country show an increase in most countries from March, a peak in April, and then a rapid decline to relatively low levels. However, the prevalence rates in the post-adjusted countries remained high compared to those in the unadjusted countries. It was easy to find that the United States was still the country with the highest prevalence rate between May and September.

**Table 1:** Actual and expected prevalence rates in critical countries from March to September 2020.

Country codes	Countries	March 2020		April 2020		May 2020		June 2020		July 2020		August 2020		September 2020	
		Actual (%)	Expected (%)	Actual (%)	Expected (%)	Actual (%)	Expected (%)	Actual (%)	Expected (%)	Actual (%)	Expected (%)	Actual (%)	Expected (%)	Actual (%)	Expected (%)
BEL	Belgium	0.13	7.92	0.30	10.90	0.08	1.55	0.03	0.21	0.07	0.23	0.14	0.33	0.21	0.42
CAN	Canada	0.02	0.23	0.12	1.99	0.10	1.41	0.04	0.31	0.03	0.09	0.03	0.07	0.05	0.14
DEU	Germany	0.07	1.04	0.12	2.27	0.03	0.39	0.02	0.10	0.02	0.05	0.04	0.08	0.04	0.08
ESP	Spain	0.22	7.28	0.24	5.22	0.05	0.67	0.02	0.36	0.08	0.17	0.37	0.74	0.47	0.95
FRA	France	0.07	5.49	0.13	7.31	0.03	1.03	0.02	0.22	0.03	0.13	0.14	0.28	0.28	0.59
GBR	England	0.04	2.68	0.21	7.20	0.13	2.44	0.04	0.48	0.03	0.13	0.05	0.10	0.10	0.21
IRN	Iran	0.05	0.63	0.06	0.42	0.07	0.34	0.09	0.71	0.09	1.06	0.09	0.74	0.07	0.64
ITA	Italy	0.17	4.99	0.17	3.32	0.05	0.86	0.01	0.16	0.01	0.08	0.03	0.09	0.05	0.11
JPN	Japan	0.00	0.01	0.01	0.11	0.00	0.04	0.00	0.00	0.01	0.03	0.03	0.07	0.01	0.03
KOR	Korean	0.01	0.05	0.00	0.01	0.00	0.00	0.00	0.01	0.00	0.01	0.01	0.02	0.01	0.02
NLD	Netherlands	0.07	3.58	0.16	5.05	0.04	0.81	0.02	0.12	0.02	0.05	0.09	0.19	0.16	0.33
RUS	Russia	0.00	0.00	0.07	0.18	0.20	0.54	0.17	0.55	0.13	0.45	0.11	0.35	0.09	0.31
SGP	Singapore	NA	NA	0.25	0.57	0.32	0.70	0.16	0.34	0.14	0.30	0.09	0.18	0.01	0.03
USA	America	0.05	0.64	0.27	2.80	0.22	1.37	0.25	0.79	0.58	1.40	0.46	1.17	0.27	0.67

### Actual and expected imported cases

Table 2 shows the actual and expected imported cases. It can find that the real imported cases were higher from March to April in 2020, and then there was a significant decrease. There was a particular rebound in August, but it showed a downward trend in September. If no airline control was carried out, there were many imported cases in March and April, which peaked in April. Then the imported cases fluctuated, but they also remained at a large number, so if there were no flight restrictions and control, it could lead to a severe secondary outbreak.

**Table 2:** Actual and expected importing cases in China from March to September 2020 (number of individuals).

Time	Actual importing cases	Expected importing cases without airline control
Mar-20	787	15,794
Apr-20	750	34,033
May-20	88	15,711
Jun-20	170	8414
Jul-20	138	10,233
Aug-20	428	11,533
Sep-20	355	6881

### Analysis of expected imported cases in each critical importing country

Since March 2020, the world epidemic has spread rapidly, with a high overall expected number of imported cases and a high number of importing countries (Table 3). The top 10 expected importing countries among them in descending order of the number of imported cases were called critical importing countries, namely France (FRA 2762), Spain (ESP 2598), Italy (ITA 2567), the United States (USA 1941), the United Kingdom (GBR 1720), Germany (DEU 847), Belgium (BEL 756), Netherlands (NLD 562), Korea (KOR 322), and Switzerland (CHE 303).

The rapid increase in expected imported cases continued in April 2020, with the United States (USA 7840), United Kingdom (GBR 5543), France (FRA 4955), Italy (ITA 2134), Spain (ESP 2060), Germany (DEU 1916), Canada (CAN 1697), Singapore (SGP 1548), Netherlands (NLD 1162), and Belgium (BEL 1141), the top 10 critical importing countries were all expected to import more than 1000 cases, with a continued rapid growth rate compared to March.

The overall expected number of imported cases in May 2020 was somewhat lower than in April, with the United States (USA 5667), Singapore (SGP 1904), the United Kingdom (GBR 1876), Canada (CAN 1142), France (FRA 712), Russia (RUS 672), Italy (ITA 545), the United Arab Emirates (ARE 351), Germany (DEU 336) and Japan (JPN 313), with still four critical importing countries with more than 1000 cases.

The expected number of imported cases in June 2020 decreased steadily compared to May; only the United States (USA 3111) was more than 1000 cases. Other critical importing countries were Singapore (SGP 944), Russia (RUS 862), United Kingdom (GBR 526), Canada (CAN 308), Spain (ESP 194), Brazil (BRA 178), United Arab Emirates (ARE 169), France (FRA 166), and Switzerland (SWE 156).

The number of expected imported cases continued to decline in most countries in July 2020. Still, the number of expected imported cases in the United States continued to show a significant increase. The top 10 countries in terms of expected imported cases were the United States (USA 4812), Singapore (SGP 886), Russia (RUS 824), Japan (JPN 225), Indonesia (IDN 222), the United Kingdom (GBR 200), Brazil (BRA 186), Australia (AUS 177), South Africa (ZAF 175), and the Philippines (PHL 155). Some new countries emerged among the top 10 countries, such as Indonesia, Australia, South Africa, and the Philippines.

From August 2020, the number of expected imported cases gradually decreased in most countries that ranked high in the previous months. Still, the number of expected imported cases increased further in the new countries that entered the top 10, such as Australia, the Philippines, Indonesia, etc. The top 10 countries and the number of imported cases in August were as follows: United States (USA 4470), Russia (RUS 677), Japan (JPN 638), Singapore (SGP 580), Australia (AUS 530), Philippines (PHL 425), Maldives (MDV 381), Spain (ESP 355), Indonesia (IDN 257), and France (FRA 234).

In September 2020, the expected number of imported cases declined in the formerly top-ranked critical importing countries. However, there was still a steady increase in the bottom-ranked countries such as France, Spain, Indonesia, and the United Arab Emirates. The specific situation was as follows: United States (USA 1889), Russia (RUS 528), France (FRA 414), Spain (ESP 362), the Philippines (PHL 233), Israel (ISR 231), Indonesia (IDN 229), the United Arab Emirates (ARE 227), and Japan (JPN 206).

**Figure 4:**
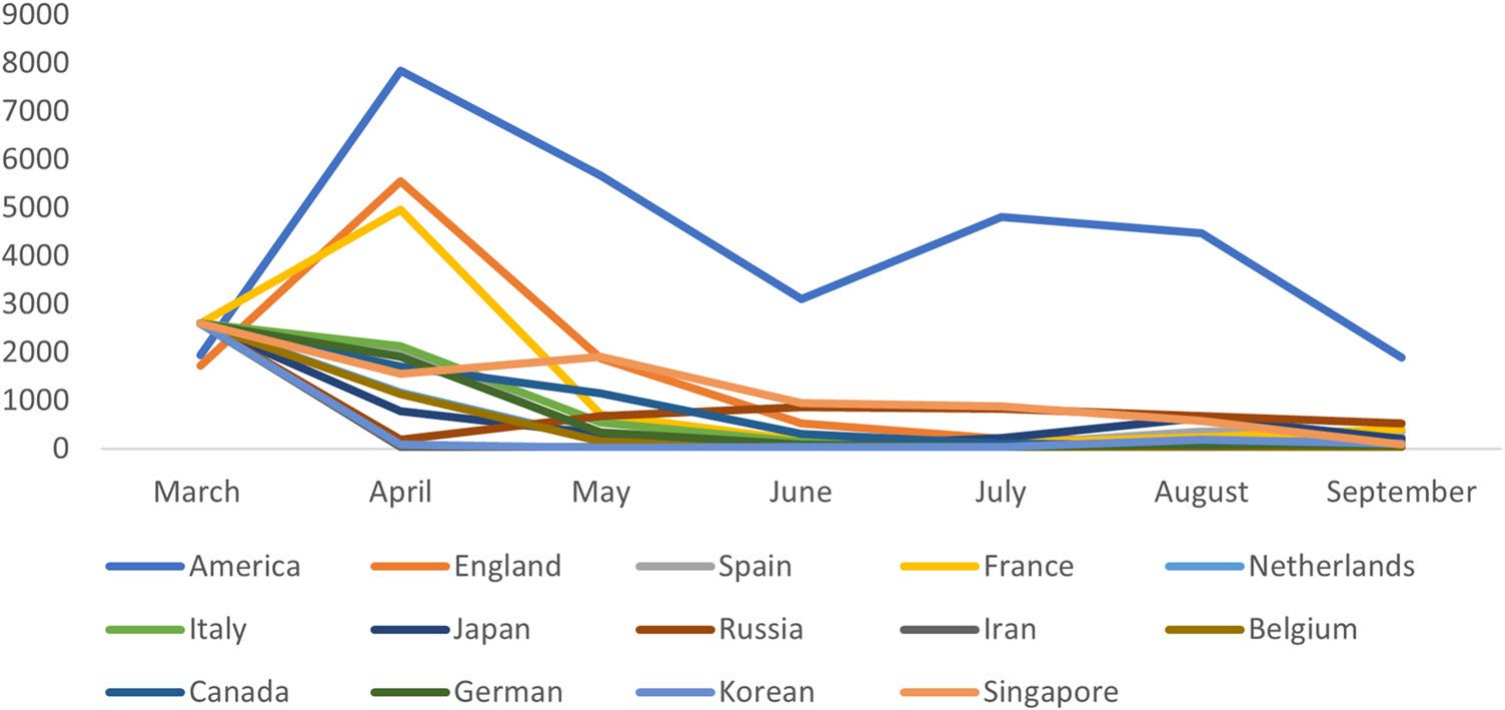
Expected number of imported cases in critical countries from March to September 2020.

The change situation of the expected number of imported cases in each critical importing country from March to September 2020 is shown in Fig. 4. From March to May 2020, the expected imported cases were mainly concentrated in North America and countries in the European region, primarily the United States, the United Kingdom, France, Italy, Singapore, Germany, Canada, and the Netherlands. From June to September, the expected imported cases fluctuated. The expected cases in many East Asian countries gradually increased, accounting for a more significant part of the total predicted imported cases. The U.S. had been ranked first since April with a much higher number of expected imported cases than the country with the second-highest number of expected imported cases. The countries with more actual imported cases were Russia, the United Kingdom, the United States, the Philippines, and Spain. It can be found that the top 10 countries in terms of actual imported cases were not all the same as the top 10 countries in terms of expected imported cases. There was a significant increase in the number of expected imported cases compared to the actual ones. A large number of imported cases would be generated without the control and lockdown of flights, thus then causing a rebound or even a secondary outbreak of the disease.

**Table 3:** Actual and expected imported cases in critical countries from March to September 2020.

Countries	March 2020		April 2020		May 2020		June 2020		July 2020		August 2020		September 2020	
	Actual	Expected	Actual	Expected	Actual	Expected	Actual	Expected	Actual	Expected	Actual	Expected	Actual	Expected
America	115	1941 (835-4147)	49	7840 (3428–16618)	13	5667 (2439–12069)	13	3111 (1321–6576)	21	4812 (2218–9955)	18	4470 (1934–9913)	21	1890 (857–3987)
England	222	1721 (872–3182)	80	5543 (2862–10779)	4	1876 (957–3610)	5	526 (245–964)	4	200 (96–386)	28	176 (92–405)	5	238 (130–460)
Spain	78	2599 (1346–4706)	5	2060 (1003–4035)	1	279 (132–802)	2	194 (50–482)	0	97 (46–199)	5	355 (197–592)	7	363 (198–818)
France	6	2762 (1398–5075)	0	4956 (2554–9264)	0	713 (331–1373)	0	167 (73–337)	0	119 (52–247)	0	234 (127–427)	0	414 (223–757)
Netherlands	3	563 (281–1047)	3	1163 (580–2164)	0	185 (90–355)	0	26 (11–56)	2	12 (6–21)	0	39 (21–65)	0	68 (38–117)
Italy	48	2568 (1332–4675)	2	2134 (1109–3907)	0	545 (278–996)	0	132 (64–248)	0	74 (35–144)	0	85 (43–160)	1	71 (39–118)
Japan	2	80 (35–168)	1	782 (372–1540)	4	313 (144–633)	0	39 (18–81)	0	225 (120–400)	4	638 (299–1256)	2	206 (87–429)
Russia	33	5 (1–10)	532	190 (80–367)	30	672 (323–1262)	6	863 (387–1620)	20	824 (374–1553)	10	678 (315–1271)	5	528 (241–1003)
Iran	43	48 (24–89)	1	42 (21–79)	0	39 (19–72)	1	68 (34–126)	1	107 (53–199)	0	78 (39–145)	2	67 (33–125)
Belgium	2	756 (364–1442)	1	1141 (555–2185)	0	157 (70–306)	0	23 (10–47)	0	33 (13–69)	0	33 (15–66)	1	40 (22–68)
Canada	8	197 (96–374)	5	1700 (848–3180)	1	1143 (573–2137)	2	308 (147–597)	1	98 (46–199)	3	76 (41–136)	2	127 (62–245)
German	6	847 (424–1595)	0	1916 (960–3602)	0	336 (166–639)	0	80 (37–159)	0	43 (21–90)	0	84 (46–147)	0	77 (43–132)
Korean	0	323 (147–667)	0	82 (34–183)	0	26 (11–61)	0	39 (20–75)	0	43 (23–75)	1	185 (95–356)	3	113 (49–266)
Singapore	2	NA	1	1548 (761–5430)	1	1904 (972–4770)	1	944 (492–2086)	11	886 (463–1910)	40	580 (302–1288)	13	82 (42–215)
United Arab Emirates	10	15 (6–38)	1	675 (295–1460)	4	351 (170–715)	4	169 (94–292)	0	146 (81–266)	53	177 (64–222)	7	227 (125–397)
Indonesia	3	26 (12–50)	0	62 (30–121)	0	94 (46–178)	4	144 (71–271)	4	222 (111–418)	17	257 (128–483)	25	229 (114–432)
The Philippines	42	34 (16–73)	1	67 (30–146)	2	34 (14–100)	1	55 (25–141)	24	155 (79–332)	87	425 (213–907)	70	233 (105–592)

### Effective reproduction number

**Figure 5:**
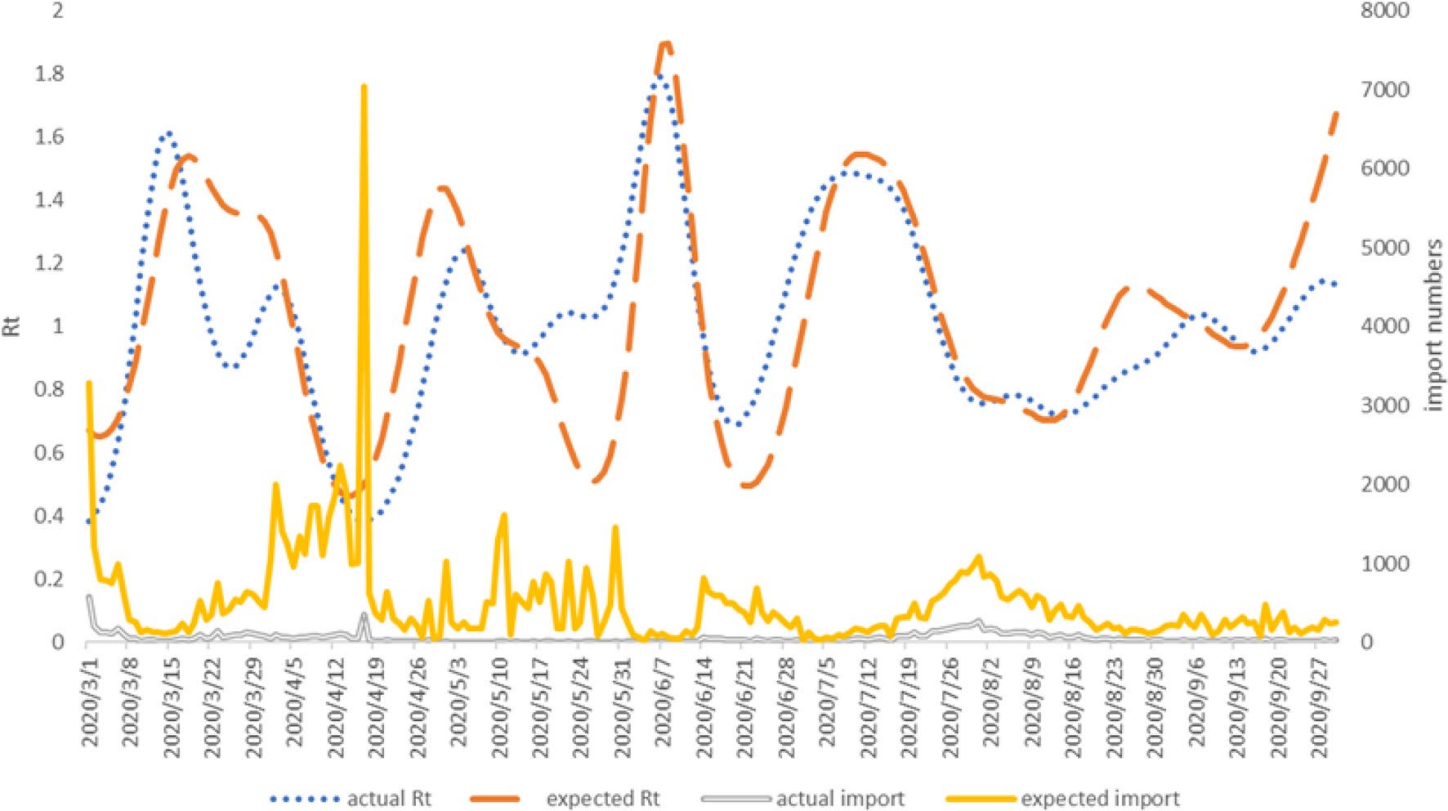
Acutal Rt and expected Rt with actual and expected number of importing cases in 2020.

Figure 5 shows the estimated actual and expected time-varying $R_t$ with the actual and estimated number of reported cases. It can be seen that the fluctuation between time-varying $R_t$ and the number of reported cases was closely correlated at inevitable delays.

The actual $R_t$ change showed a rapid increase from the beginning of March 2020, reaching 1.61 (90% CI (1.35, 1.88)) in the second week (March 15th), followed by a decline to 0.89 (90% CI (0.69, 1.05)) in the middle of the third week (March 25th). The actual $R_t$ slightly increased to 1.09 (90% CI (0.90, 1.31)) in the fifth week (April 4th) and then dropped sharply to the beginning of the study of 0.38 (90% CI (0.27, 0.49)). It had a relatively large increase to 1.23 (90% CI (0.97, 1.54)) in the 9th week (May 5th); followed by smaller fluctuations, including a drop to 0.91 (90% CI (0.67, 1.14)) in the middle of week 10 (May 14th) and an increase to 1.04 (90% CI (0.83, 1.29)) in week 12 (May 23rd). The actual $R_t$ had a larger increase, reaching a peak of 1.79 (90% CI (1.42, 2.19)) in week 14 (June 7th); followed by a large decline to 0.69 (90% CI (0.52, 0.86)) in week 16 (June 20th). Then it rose to 1.48 (90% CI (1.27, 1.75)) in week 19 (July 10th); fell to 0.76 (90% CI (0.61, 0.90)) in week 21 (August 1st) and rose, after minor fluctuations, to 0.78 (90% CI (0.63, 0.95)) in week 22 (August 7th). The actual $R_t$ decreased to 0.72 (90% CI (0.56, 0.88)) in week 23 (August 15th); then increased with fluctuations to 1.04 (90% CI (0.85, 1.27)) in week 26 (September 7th). It decreased to 0.92 (90% CI (0.75, 1.09)) in the middle of week 27 (September 17th) ), and rose to 1.14 (90% CI (0.92, 1.38)) in week 30 (September 29th). It can be seen that the period of each fluctuation was basically around 2-3 weeks until week 15. Then the fluctuation period became gradually longer, the range became smaller, and the value fluctuated around 1.

The expected $R_t$ change showed a rapid rise from March 2020, reaching 1.58 (90% CI (1.22, 1.70)) in week 2 (March 15th). It declined slightly to 1.36 (90% CI (1.13, 1.59)) in the middle of week 3 (March 26th), followed by a rapid decline to 0.47 (90% CI (0.35, 0.58)) in the middle of week 6 (April 15th). Then the expected $R_t$ rose sharply to 1.41 (90% CI (1.17, 1.65)) in week 9 (May 2nd), fell slightly to 0.93 (90% CI (0.78, 1.10)) in the middle of week 10 (May 14th). It fell further to 0.51 (90% CI (0.35, 0.67)) in week 12 (May 26th) and increased sharply to 1.90 (90% CI (1.51, 2.37)) in week 14 (June 8th), reaching the highest value in the study range. The expected $R_t$ decreased sharply to 0.51 (90% CI (0.36, 0.65)) in week 16 (June 23rd), then increased in week 19 (July 13th) to 1.54(90% CI (1.27, 1.78)). It declined to 0.79 (90% CI (0.62, 0.97)) in mid-week 21 (July 31st), then continued to decline slowly to 0.71 (90% CI (0.56, 0.86)) in mid-week 23 (August 13th). The expected $R_t$ increased slightly to 1.13 (90% CI (0.92, 1.37)) in mid-week 25 (August 27th); then declined slightly to 0.94 (90% CI (0.76, 1.14)) in week 28 (September 15th); and then showed an increasing trend until the end of the study, reaching 1.67 (90% CI (0.46, 4.01)) in week 30 (September 30th). The trend was similar to the actual $R_t$, with 2-3 weeks fluctuation until week 16, followed by a longer fluctuation period and a slighter range. The difference was that there was a delay in the expected $R_t$ compared with the actual $R_t$. Each fluctuation was slightly different because the $R_t$ was related to daily diagnosed cases and all previous dates. Also, the estimation of expected $R_t$ took into account the latency period and the time of reporting delay.

Paired ranked one-tailed test was used to compare the estimates of expected time-varying $R_t$ with those of actual time-varying $R_t$. It found that the predicted time-varying $R_t$ was significantly greater than the actual time-varying $R_t$ (V = 13828, p-value = 0.0052). It indicated that the aviation control measures implemented since the outbreak of COVID-19 had effectively prevented the spread of the epidemic in China.

## Discussion

This research predicted the number of imported cases without aviation control via evaluating the situation of COVID-19 prevalence in the importing country and the data of flights among specific countries. When estimating the prevalence rate, the number of actual reported cases calculated by the real prevalence rate was identical to the result of the accumulated number of reported cases in the critical imported country in the thesis, which demonstrated the accuracy and stability of the calculation of the prevalence rate^24^. In addition, there might be several reasons when the variation tendency of prevalence rate was different from the reality.

First, some studies illustrated that after the first pandemic of COVID-19, the diagnosis rate in many countries decreased. For example, in France, according to the model of health recording, although there were surveillance and control measures, many infected people did not receive testing or quarantine, which led to a low diagnosis rate^25,26^. Therefore, the proportion we estimated from March to April in 2020 was relatively higher than the other months, resulting in a rapid increase in the adjusted number of cases. However, the proportion of undiagnosed cases decreased after May 2020. Then the number of adjusted cases decreased but was also relatively higher than the actual reported number of cases.

In addition, as mentioned above, the baseline CFR was assumed as 1.4% (95% CI 1.2–1.5%). Meanwhile, the range of asymptomatic proportion was assumed by 50% (95% CI 10–70%). Considering the uncertainty of the estimated prevalence rate value in the inference process, we propagated the variance of the baseline CFR, so the final 95% credible interval reported for under-ascertainment reflects underlying uncertainty in the model parameters. Thus, the ultimate 95% confidence interval would reflect the potential uncertainty of the model’s parameter. Our estimates were consistent with published serological data, which to some extent demonstrated the stability of estimation^27^. At the same time, there were some restrictions because we assumed that the death case report was accurate. If the ability to identify was limited or there were some other factors affecting the death, some death cases may be incorrectly attributed to other death reasons. In this situation, we may underestimate the infection rate.

Furthermore, CFR in some countries may concentrate on a specific age group, and effective CFR may be either higher or lower than the baseline CFR we preset. So if we could group the baseline CFR according to different countries and ages, we could measure the disease burden in every country more precisely. In our paper, we simplified the explanation and calculation by presetting a reasonable range of CFR.

The results of calculating expected imported cases on foreign flights visualized that theoretically expected imported cases would far exceed actual imported cases if no airline control measures were implemented. This result was validated in the relevant thesis. One study analyzed the effect of simulated scenarios with different levels of airline control on an epidemiological curve and imported cases in the country. It showed that when combined with public health interventions and behavior change, airline control can significantly reduce the spread of the disease^28^, which was the same conclusion as our analysis.

The values of time-varying $R_t$ estimated in this paper were similar to the results of other theses on time-varying $R_t$ in Asian countries^29,30^, both exhibiting relatively low $R_t$. Due to effective control measures in China making $R_t$ fluctuate around 1, the transmission rate of COVID-19 in China was relatively low. The approach for estimating time-varying $R_t$ had many advantages. First, the estimation of the $R_t$ used only the number of reported cases, which enabled our method to be applied to a broader range of scenarios and to be able to be used in studies that provide different types of data. For example, we can use the corresponding delayed time distributions in other kinds of data providing the number of inpatients, deaths, et al.. In addition, calculating confidence intervals with this method was more straightforward and accessible than using maximum likelihood estimation methods to estimate $R_t$^31^. It was also easier to implement and more flexible than parameter estimation used on the same data set^32^.

Of course, this method had some limitations. This method required the proportion of reported infections to remain constant over the study time. In other words, it needed consistency in case collecting, the intensity of nucleic acid testing, and the definition of cases. However, the intensity of nucleic acid testing and the extent of delayed reporting in a country will often vary as the pandemic progresses. But if this variation remains consistent after the start, it will only temporarily affect the estimated value. If the actual testing capacity was limited, or if there were some special distributions of cases in the early stage, we may underestimate the actual burden of the infection. The duration of bias in the $R_t$ estimates will depend on the length of the incubation period of the case, the delayed distribution of reports, and the parameter settings of the Gaussian process used in the $R_t$ estimation process. The impact of nucleic acid testing and other reporting biases on $R_t$ depended on the measures used to control transmission (number of test-positive cases, number of hospitalizations, number of test-positive deaths). In addition, the delay from episodes to reporting affected the number of cases calculated by the date of episodes. If the actual delay were shorter than our estimated delay, it would overestimate the number of cases and, conversely, underestimate it. And this delay varied with the transmission characteristics and situations of the disease. In our paper, we used a re-sampling approach to generate distributions of delay times to reduce the uncertainty of such variation.

What’s more, adjusting for the actual number of asymptomatic was performed by simply assuming a wide range, reflecting the still-present uncertainty in the literature of 10-70% of all infections. The proportion of asymptomatic infections has been estimated to also vary with age^21^.In the absence of age-stratified data globally, we opt for a simple adjustment, which is equivalent across all settings. Therefore, we likely overestimate incidence in countries with younger populations and vice versa in countries with older people. As more detailed data comes in, it would be possible to refine and improve the methods’ accuracy. When considering the estimation of $R_t$, we chose an experienced approach to assume the $R_0$. Some papers mentioned that it would be a different $R_0$ when the other pandemic waves appeared, so it will be possible to consider more details on the specific pandemic situation of each country^33^.The Bayesian latent variable method was used to estimate $R_t$ in this study. Other methods, such as the Bernoulli S-I (Susceptible-Infected) equation, estimate transmission rates by the new daily infections^34^.These methods considered the epidemic’s growth and provided another way to solve this problem. Comparison between these methods will be considered in the future.

In conclusion, the analysis of the effects of aviation controls on the COVID-19 epidemic in the country can help evaluate and develop similar public health response plans in the future. This study showed that the aviation controls from March to September 2020 played an essential role in controlling the development of the epidemic in China.

## Data Availability

Correspondence and requests for data and materials should be addressed to authors.
